# A serum-circulating long noncoding RNA signature can discriminate between patients with clear cell renal cell carcinoma and healthy controls

**DOI:** 10.1038/oncsis.2015.48

**Published:** 2016-02-15

**Authors:** Y Wu, Y-Q Wang, W-W Weng, Q-Y Zhang, X-Q Yang, H-L Gan, Y-S Yang, P-P Zhang, M-H Sun, M-D Xu, C-F Wang

**Affiliations:** 1 Department of Pathology, Fudan University Shanghai Cancer center, Shanghai, China; 2 Department of Oncology, Shanghai Medical College, Fudan University, Shanghai, China; 3 Institute of Pathology, Fudan University, Shanghai, China; 4 Department of Pathology, Obstetrics and Gynecology Hospital of Fudan University, Shanghai, China; 5 Department of Pathology, Rui Jin Hospital, Shanghai Jiao Tong University School of Medicine, Shanghai, China

## Abstract

Serum biomarkers have not been fully incorporated into clinical use for the diagnosis of renal cell carcinoma (RCC). The recent discovery of long noncoding RNAs (lncRNAs), which have been reported in a variety of cancer types, suggested a promising new class of biomarkers for tumour diagnosis. The aim of our study was to evaluate whether the levels of circulating lncRNAs could be used as a tumour marker to discriminate between clear cell RCC (ccRCC) patients and healthy controls. Serum samples were collected from 71 ccRCC patients including 62 age- and sex-matched healthy controls and 8 patients with benign renal tumours. Eighty-two cancer-associated lncRNAs were assessed by reverse transcription and quantitative polymerase chain reaction in paired tissues and serum. A 5-lncRNA signature, including lncRNA-LET, PVT1, PANDAR, PTENP1 and linc00963, were identified and validated in the training set and testing set, respectively. The receiver operating characteristic curves for this serum 5-lncRNA signature were 0.900 and 0.823 for the two sets of serum samples. Moreover, five-minus-one lncRNA signatures demonstrated that none of the lncRNAs had a higher area under the curve than the others in either set. A risk model for the serum 5-lncRNA signature also determined that benign renal tumours can be distinguished from ccRCC samples. This work may facilitate the detection of ccRCC and serve as the basis for further studies of the clinical value of serum lncRNAs in maintaining surveillance and forecasting prognosis.

## Introduction

Renal cell carcinoma (RCC) is 1 of the 10 most common cancers, with approximately 202 000 cases and 102 000 deaths worldwide.^1, 2^ The incidence of RCC has increased for over two decades,^3^ and most tumours are asymptomatic and nonpalpable in early stages. Clear cell RCC (ccRCC) is the most common subtype and represents approximately 70% of all renal tumours.^4^ Metastasis is common in ccRCC, and approximately one-third of ccRCC patients have metastasis at the time of diagnosis despite the wide use of ultrasound and computed tomography. Thus, effective tools for the early detection of ccRCC are critically necessary.

The identification and characterization of the genetic changes that drive renal cancer development and progression have provided us with a variety of molecular markers,^5^ such as the mutation of the VHL gene. However, these markers have not been fully adapted for clinical use for diagnosis either because they lack sensitivity or because the molecular assays are too cumbersome. Ideally, biomarkers should be easily accessible and sampled noninvasively. Circulating cell-free nucleic acids have attracted much interest.^6, 7, 8^ Previous reports have confirmed the presence of microRNAs (miRNAs) in serum and have demonstrated that circulating miRNAs with diagnostic potential exist for almost every type of malignancy.^9, 10, 11^ However, inconsistent results restricted the clinical use of circulating miRNAs. Recently, long noncoding RNAs (lncRNAs), which are a newly discovered class of noncoding RNAs (ncRNA) >200 nucleotides in length, have been increasingly reported in a variety of cancer types, suggesting an important role in tumourigenesis and also implying a promising new class of biomarkers for tumour diagnosis.^12^

However, there have been few systematic reports on the role of circulating lncRNAs in ccRCC. In this study, we selected 82 cancer-associated lncRNAs (additional file 2 Supplementary Table S1) from the LncRNADisease database (http://cmbi.bjmu.edu.cn/lncrnadisease) and evaluated their expression in tissues and serum. The purpose of our study was to determine whether the circulating lncRNAs could discriminate ccRCC patients from age- and sex-matched healthy controls. In addition, the potential relationship between circulating lncRNA levels and the clinicopathological features of ccRCC was investigated.

## Results

### Patient characteristics

The sera from a total of 141 participants, including 71 ccRCC patients, 62 healthy controls and 8 patients with benign renal tumours, were entered into this study. Table 1 lists the clinical characteristics and pathological information in the training set, testing set and additional set, excluding the phase of marker discovery. Among the 71 ccRCC cases, the maximum tumour diameter was ⩽ 4 cm in 16 patients (22.5%); 28 patients were diagnosed as stage I (39.4%), and the Fuhrman grading system showed that only 2 cases were grade 1, whereas 21 (29.6%), 30 (42.3%) and 8 (11.3%) cases were grades 2, 3 and 4, respectively. Lymph nodes metastasis, vascular invasion and distant metastasis accounted for 32.4, 26.8 and 14.1% of cases in the ccRCC patients.

### Discovery of candidate lncRNAs in tissues

At the beginning of this study, the expression levels of the 82 lncRNAs in RCC and adjacent non-malignant tissues (including 12 ccRCCs, 7 chromophobe RCCs and 6 PRCCs) were determined using reverse transcription (RT)–PCR. LncRNA expression was normalized to β-actin as described in the literature^13, 14^ and the mean expression level was calculated. We then compared lncRNA tissue profiles to identify potential lncRNAs that could serve as diagnostic biomarkers. The criteria for further investigation of these selected candidates were: (1) different expression (*P*<0.05) and (2) quantification cycle values < 30 to enable reliable detection. Based on these criteria, 31 cancer-associated lncRNAs were chosen for the next phase (additional file 2 Supplementary Table S1, additional file 1 Supplementary Figure S1).

### Establishing the predictive lncRNAs panel

**Figure 1:**
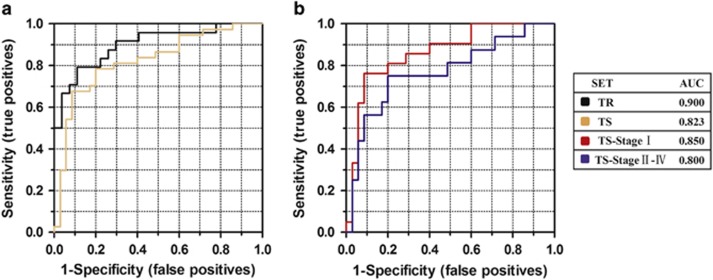
The serum 5-lncRNA diagnostic model. Receiver operating characteristic (ROC) curves of the 5-lncRNA diagnostic model in the training set (TR) and the testing set (TS). The AUC values of the serum 5-lncRNA signature of both sets provided the greatest predictive ability (**a**). The predictor also performed well for cancers of all stages in TS when divided into TS—stage I and TS—stages II–IV (**b**).

**Figure 2:**
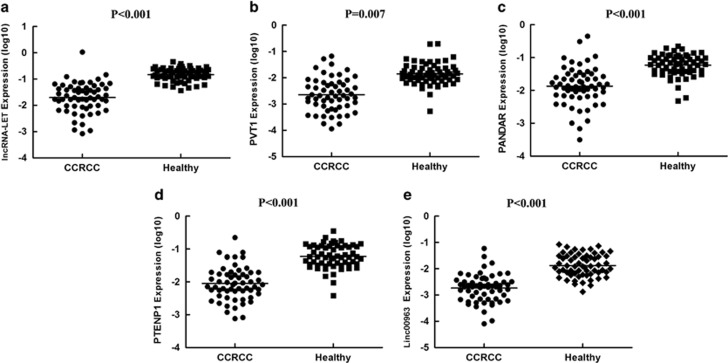
Distribution of lncRNA-LET (**a**), PVT1 (**b**), PANDAR (**c**), PTENP1 (**d**) and linc00963 (**e**) levels from the serum of patients and healthy controls in the training set by RT–qPCR.

After marker discovery, we used the training set to detect the levels of these promising lncRNAs by RT and quantitative polymerase chain reaction (RT–qPCR) in a cohort of 24 patients with ccRCC and 27 normal controls. Using β-actin as a normalization control, 9 lncRNAs with a detection rate of <75% (such as CDKN2B-AS1, SNHG5, LSINCT5 and AK126698) and 3 lncRNAs with a Cq value of >35 (HIFA-AS2, ZFAS1 and CCAT1) were excluded from further analysis. In addition, five lncRNAs (AS1DHRS4, TUG1, XIST, DLEU1 and PCAT1) with a *P*-value of >0.05 were excluded. Consequently, 13 significantly downregulated lncRNAs and 1 significantly upregulated lncRNA (MALAT1) were identified in the sera from the ccRCC patients. Then, a stepwise selection model revealed that the combination of lncRNA-LET, PVT1, PANDAR, PTENP1 and linc00963 (additional file 2 Supplementary Table S2, additional file 1 Supplementary Figure S2) provided the greatest predictive ability, with an area under the curve (AUC) of 0.90 (95% confidence interval: 0.814–0.986) (Table 2, Figure 1a), under the condition that the AUC value of a single lncRNA (additional file 2 Supplementary Table S3) was lower than that of the 5-lncRNA signature. The differential expression levels of the five lncRNAs are shown in Figure 2.

### Testing set of the lncRNA panel

**Figure 3:**
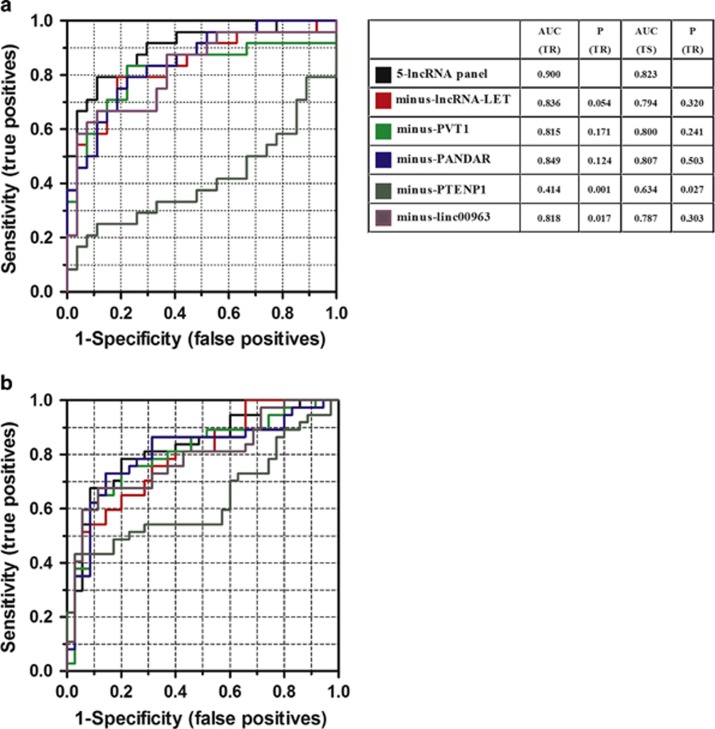
Receiver operating characteristic (ROC) curves of the 5-lncRNA panel (**a**) and five-minus-one lncRNA signatures (**b**) in the training set (TR) and the testing set (TS). Comparative ROC was determined by the 5-lncRNA panel and the other five-minus-one lncRNA signatures. The AUC and *P*-value are listed in the picture.

We thus validated a 5-lncRNA panel using the same method in a test cohort of 37 patients with ccRCC and 35 healthy controls. The predictor was remarkably stable, with an AUC of 0.823 (Table 2, Figure 1a). In addition, it is noteworthy that although we divided the testing set by TNM stage, the predictor performed well for cancers of all stages (I, II–IVs), with an AUC of 0.85 for stage I tumours and 0.80 for stages II–IV tumours (Table 2, Figure 1b), supporting its ability to detect ccRCC patients at all stages, particularly for early-stage tumours. To confirm that these five lncRNAs were essential for the 5-lncRNA signature, we also constructed five-minus-one lncRNA signatures by deleting each lncRNA one at a time and comparing the AUCs of these four lncRNA signatures with the original 5-lncRNA signature. Unlike the 5-lncRNA signature, none of the five-minus-one lncRNA signatures had a higher AUC in the training set and testing set (Figure 3).

### Additional set of clinical validation

**Figure 4:**
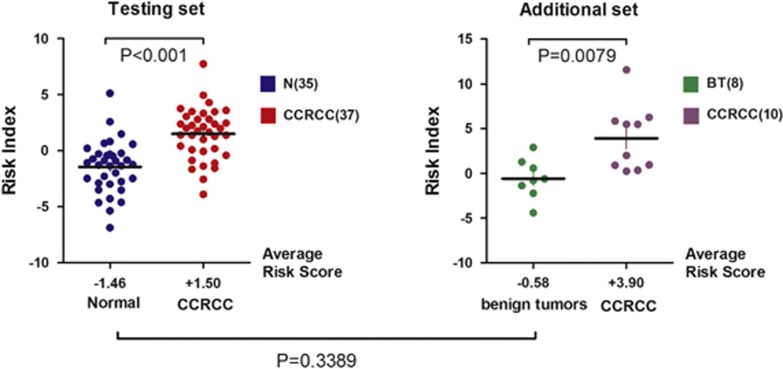
Risk of cancer based on the 5-lncRNA risk model in ccRCC patients from the testing set (left) and the additional set (right). The average risk scores and *P*-values (ANOVA) are also shown.

Finally, we further analyzed the 5-lncRNA predictor to gain further insights into its potential value in the clinical setting. Ten independent serum ccRCC subjects and eight benign serum renal tumour subjects were used. When the 5-lncRNA predictor was applied to evaluate the risk in the ccRCC and benign tumour (BT) set, it performed remarkably well (Figure 4). The average risk index of ccRCC patients from the additional set was clearly statistically significantly higher than that of the BT set (average risk score of ccRCCs=3.90, BT=−0.58, *P*-value=0.0079 between ccRCCs and BT; Figure 4, right). In addition, the risks of the normal individuals from the testing set and of the BT patients from the additional set were similar, and the difference was not statistically significant (testing set normal=−1.46, additional set BT=−0.58, *P*=0.339; Figure 4).

### Correlation with clinical outcomes

The analysis results of the correlation between serum panel expression levels and clinical parameters are only for the samples in the training and testing set; the additional set was not included. The relative expression level of the panel of five lncRNAs was calculated using the regression equation generated by the stepwise regression analysis. As shown in Table 1, only age (Pearson's *χ*^*2*^ test) was significantly associated with the panel.

## Discussion

In routine clinical practice, RCC is solely diagnosed by imaging examinations, such as ultrasound and computed tomography. Compared with other cancers, there are very few tumour biomarkers for renal cancer.^15^ Previous studies have described the potential use of circulating nucleic acids, including DNA^16, 17^ and miRNAs,^18, 19, 20^ as non-invasive biomarkers for ccRCC. However, for the diagnosis of RCC, few highly sensitive or specific tumour markers are available. LncRNA, which is an emerging class of ncRNA, has demonstrated functions in the regulation of chromatin structure, gene expression and translational control. Many recent studies have described the expression profile of lncRNAs in tissues and cell lines. For example, Hirata *et al.*^21^ demonstrated that MALAT1 is markedly increased in RCC tissues and cell lines and that the overexpression of MALAT1 promotes aggressive RCC through Ezh2 and interacts with miR-205. Bertozzi *et al.*^22^ determined that HIF-1alpha-AS1 and AS2 could be used to stratify renal cancer by subtype based on the expression level. The reliance on surgical resection, which is an invasive procedure for tissue sample collection, limits the application in cancer diagnosis. Research on serum lncRNAs, which are relatively easy to access, is exceedingly rare. However, as indicated in literatures, previous data about tissues lncRNA expression in RCC has been reported. Fachel *et al.*^23^ demonstrated that a signature of 29 intronic lncRNAs differentially expressed between RCC and nontumour samples through combining microarray experiments and large-scale public data. They also found a signature of 26 intronic lncRNAs significantly correlated with the RCC 5-year patient survival outcome. Malouf *et al.*^24^ and Blondeau *et al.*^25^ identified many novel lncRNA transcripts dysregulated in ccRCC successively through different methods, which may be useful for novel diagnostic biomarkers. In this study, we have systematically determined the expression levels of 91 cancer-associated lncRNA molecules in sera from ccRCC patients and established a 5-lncRNA signature as a potential marker for discriminating ccRCC patients from healthy controls.

As an initial phase of marker discovery, we used tissues to identify potential candidates. However, the results from the tissue samples were inconsistent with the RT–qPCR results obtained from individual serum samples, such as those for MALAT1, GAS5 and KCNQ1OT1, which showed significant differences in tissues, whereas the serum samples showed a detection rate of <50% or no differences. Based on these findings, the screening stage was followed by two phases of RT–qPCR, one each in the training set and the testing set. Using this approach, five significantly altered lncRNAs (lncRNA-LET, PVT1, PANDAR, PTENP1 and linc00963) were identified by a stepwise selection model. Our results showed that the 5-lncRNA panel was highly indicative of the ccRCC diagnosis. The AUC values of this 5-lncRNA panel for distinguishing ccRCC patients from healthy controls were 0.900 and 0.823. It is noteworthy that this 5-lncRNA panel had the potential to separate stage I ccRCC patients from controls (AUC=0.850), suggesting that the panel could predict ccRCC at a relatively early stage.

To further evaluate the clinical potential of this panel as a tumour marker, we established five-minus-one lncRNA signatures, which confirmed the necessity of all five lncRNAs in the panel for the diagnosis of ccRCC. We also demonstrated that the difference in the risk model between benign renal tumours and ccRCC is statistically significant, and BTs have no differences compared with normal controls. Interestingly, we found that some lncRNAs could distinguish subtypes of RCC in tissues. The expression of some lncRNAs in ccRCC patients is different from that in non-ccRCC patients, including papillary RCC and chromophobe RCC (unpublished data), suggesting a promising method for differential diagnosis between different subtypes of RCC in clinics.

Although it is established that lncRNAs in serum or plasma are quite stable and readily detected by RT–qPCR,^26^ the underlying mechanisms are unclear. It is possible that lncRNAs are protected by extracellular vesicles, including apoptotic bodies, microvesicles and exosomes^27, 28, 29^ and by complex formation with proteins, similar to what has been observed for circulating miRNAs.^30^ More recently, some studies have reported that all five of these lncRNAs are associated with tumourigenesis and the development of tumours, and our findings may better elucidate their function as markers for monitoring tumours.

LncRNA-LET is reported to be downregulated in many types of tumour tissues, including cancers of the gallbladder,^31^ and liver.^32, 33^ Yang *et al.* ^33^ demonstrated that hypoxia-induced histone deacetylase 3 repressed lncRNA-LET by reducing the histone acetylation-mediated modulation of the lncRNA-LET promoter region. Low lncRNA-LET expression was found to be associated with metastasis in clinical hepatocellular carcinoma samples. PVT1 is a widely reported oncogene,^34^ and it may be involved in colorectal cancer,^35^ gastric cancer^36^ and hepatocellular carcinoma.^37^ The function of PANDAR and linc00963 in the development of cancers had not been studied completely until recently. PANDAR's biological functions in tumours are controversial. It is downregulated in non-small cell lung cancer^38^ but upregulated in hepatocellular carcinoma.^39^ Linc00963 is only reported in prostate cancer, where it affects cellular progression.^40^ PTENP1 has been reported in the literature to have a suppressive role in cell growth by regulating cellular levels of PTEN.^41^ However, in contrast to the expression levels in tissue, PVT1 and linc00963 in serum were significantly lower in ccRCC patients than in controls. This may be because of the mechanism of secretion of circulating RNA. Some observations suggested that extracellular vesicles recruit RNA by binding to them on extracellular vesicle-contained proteins^42, 43^ and the differences between tissue and serum expression may partly depend on how much can be transferred by extracellular vesicles. In addition, serum and tissue samples from different individuals may also contribute to this phenomenon. The mechanisms accounting for the inconsistent levels of lncRNAs between tissues and serum are likely more complex in blood and must be elucidated in the future.

Taken together, we constructed a serum 5-lncRNA panel, which displays the following characteristics: (i) it can discriminate patients with ccRCC from healthy controls to facilitate diagnosis and early treatment; (ii) it will likely be considerably cheaper, easier and more immediately implementable; (iii) it requires modest amounts of serum (0.3 ml as described in this study); and (iv) it also suggests a potential use for diagnosis at an early stage. Although our observations are promising and the analytical characteristics of the 5-lncRNA panel reached values for clinical utility, large-scale prospective studies are required to verify our findings.

### Conclusion

In summary, our findings appear to provide a promising biomarker for the detection of ccRCC. This work may help patients who missed the curative treatment window benefit from early diagnosis and may also serve as the basis for future studies in personalized treatment strategies.

## Materials and methods

### Study design and patient selection

We took advantage of the LncRNADisease database with ‘cancer' as the search term, and 82 cancer-associated lncRNAs were selected and verified using the RefSeq database of the NCBI (National Centre for Biotechnology Information). We therefore designed our study to identify potential candidates among these 82 lncRNAs in 25 cancerous tissues and paired adjacent non-tumourous specimens, including 12 ccRCCs, 7 chromophobe RCCs and 6 papillary RCCs (PRCCs). Those lncRNAs that showed different expression levels were further measured in the next phase.

We then collected serum from 61 ccRCC patients undergoing radical nephrectomy, and we also investigated a control group consisting of 62 age- and sex-matched healthy subjects (men/women coming to our hospital for medical examination). These samples were further randomly divided into the training set and the testing set. The potential candidates identified above were assessed in the training set and validated in the testing set. Given that the diagnostic sensitivity and specificity of a single gene may be limited, a combination of several circulating lncRNAs was chosen as a panel of ccRCC diagnostic markers using a stepwise model selection method.

**Figure 5:**
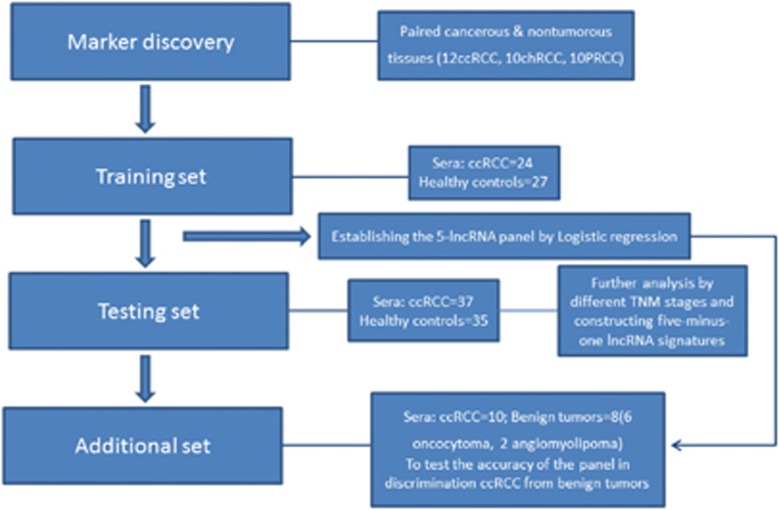
Flowchart of the study design.

Furthermore, we used an additional set of sera from 10 ccRCC patients and 8 benign renal tumours (including 6 oncocytoma and 2 angiomyolipoma) to gain further insights into the potential value of ccRCC diagnostic markers in the clinical setting. The detailed clinical–pathological parameters of 71 patients (including all ccRCC patients) were also investigated. The flowchart of these phases described above is shown in Figure 5.

This study was approved by the Ethics Committee of the Fudan University Shanghai Cancer Centre and was conducted in accordance with the tenets of the Declaration of Helsinki. The patients received the necessary information concerning the study, and their consent was obtained. Blood samples were collected before surgical operation and then centrifuged at 2800 × *g* for 10 min at 4 °C, followed by careful separation of the serum. The serum was stored at –80 °C before use. All of the patients with a renal tumour had a pathological diagnosis. RCC was graded and staged according to the Union for International Cancer Centre's Tumour Node Metastasis staging system, and the nuclear grade was evaluated by the Fuhrman criteria.

### RNA isolation

Serum RNA isolation was performed as published previously.^13^ In brief, the total RNA was extracted from 300 μl of serum using a Blood Total RNA Isolation Kit (RP4001, BioTeke, Beijing, China) and eluted in 50 μl of pre-heated (95 °C) elution solution according to the manufacturer's recommendation. The RNA quantity and purity were determined using the NanoDrop 1000 spectrophotometer (Thermo Scientific, Wilmington, DE, USA). The RNA specimens were stored at –80 °C until RT–qPCR.

### Reverse transcription and quantitative PCR

RT and qPCR kits were used to evaluate the expression levels of the selected lncRNAs. The RT reactions were performed in a volume of 50 μl using a PrimeScript RT reagent Kit (Takara, Dalian, China) and incubated for 15 min at 37 °C and 5 s at 85 °C, followed by storage at 4 °C. For real-time PCR, 1 μl of diluted-RT product was mixed with 10 μl of × SYBR Premix Ex Taq (Takara), 0.6 μl of gene-specific forward and reverse primers (10 μM), and 8.4 μl of nuclease-free water in a final volume of 20 μl according to the manufacturer's instructions. The primers used in this study are listed in additional file 2 Supplementary Table S1. All of the reactions were performed using an Eppendorf Mastercycler EP Gradient S (Eppendorf, Hamburg, Germany) with the following conditions: 95 °C for 30 s, followed by 40 cycles of 95 °C for 5 s and 60 °C for 30 s. The samples were analyzed in triplicate and included no-template controls. Amplification of the appropriate product was confirmed by a melting curve analysis following amplification. The relative expression of each lncRNA was calculated using the comparative cycle threshold (CT; 2^−ΔΔCT^) method, with β-actin (forward: 5′-TCCTCTCCCAAGTCCACACA-3′ reverse: 5′-GCACGAAGGCTCATCATTCA-3′) as the endogenous control for data normalization. The CT was defined as the number of cycles required for the SYBR signal to cross the threshold. Samples with a CT > 40 were considered negative. ΔCT was calculated by subtracting the CT values of β-actin from the CT values of the chosen lncRNA. ΔΔCT was then calculated by subtracting the mean ΔCT of the healthy control samples from the ΔCT of the ccRCC samples.

### Statistical analysis

The statistical analysis was based on the PASW statistics 18.0 (SPSS, Chicago, IL, USA). A Student's *t*-test was used to evaluate differences in the expression of the chosen lncRNAs in tissues and serum from the ccRCC patients and the corresponding controls. The sensitivity, specificity and AUC for the lncRNA levels were determined using a receiver operator characteristic analysis. Multivariate classification models were also constructed to determine the best combination of the selected serum candidates for cancer prediction. Using the binary outcome of the ccRCC serum samples and control samples (including healthy and BTs) as dependent variables, a logistic regression model was established using the stepwise model selection method. Significant differences in the average risk indexes of the various sets of patients were calculated by analysis of variance (in the case of more than two groups). The overall survival rates were analyzed using the Kaplan–Meier method with a log-rank test performed for comparison. All of the statistical tests were two sided, and a probability level of *P*<0.05 was considered statistically significant.

## Figures and Tables

**Table 1:** Correlations between serum lncRNA-LET, PVT1, PANDAR, PTENP1 and linc00963 panel expression levels and clinical parameters

	*ccRCC* (n*=71)*	*HC* (n*=62)*	*BT* (n*=8)*	P*-value*
*Sex*				0.426
Male	37	36	5	
Female	34	26	3	
*Age, years*				0.001
⩽ 60	44	41	6	
> 60	37	21	2	
*Size*				0.916
⩽ 4 cm	16			
> 4 cm	55			
*TNM stage*				0.902
I	28			
II–IV	43			
*Fuhrman grade*				0.641
G1–G2	23			
G3–G4	48			
*LN metastasis*				0.317
Yes	23			
No	48			
*Vascular invasion*				0.744
Yes	19			
No	51			
*Distant metastasis*				0.543
Yes	10			
No	61			

Abbreviations: BT, benign tumour; ccRCC, clear cell renal cell carcinoma; HC, healthy control; lncRNA, long noncoding RNA; LN, lymph node.

**Table 2:** Performance of the predictive model in various sets

*Set*	*AUC*	*ACC (%)*	*SEN (%)*	*SPE (%)*
Training set	0.9	84.1	79.2	88.9
Testing set	0.823	79.5	67.6	91.4
Testing set—stage I only	0.85	84	76.5	91.4
Testing set—stages II–IV only	0.8	80	80	80

Abbreviations: AUC, area under the curve; ACC, the overall accuracy; SEN, the sensitivity; SPE, the specificity.
