# Supplementary figures and images for: A serum-circulating long noncoding RNA signature can discriminate between patients with clear cell renal cell carcinoma and healthy controls
Source: Oncogenesis. 2016 Feb 15;5(2):e192–. doi: 10.1038/oncsis.2015.48 (PMC5154346; doi:10.1038/oncsis.2015.48)

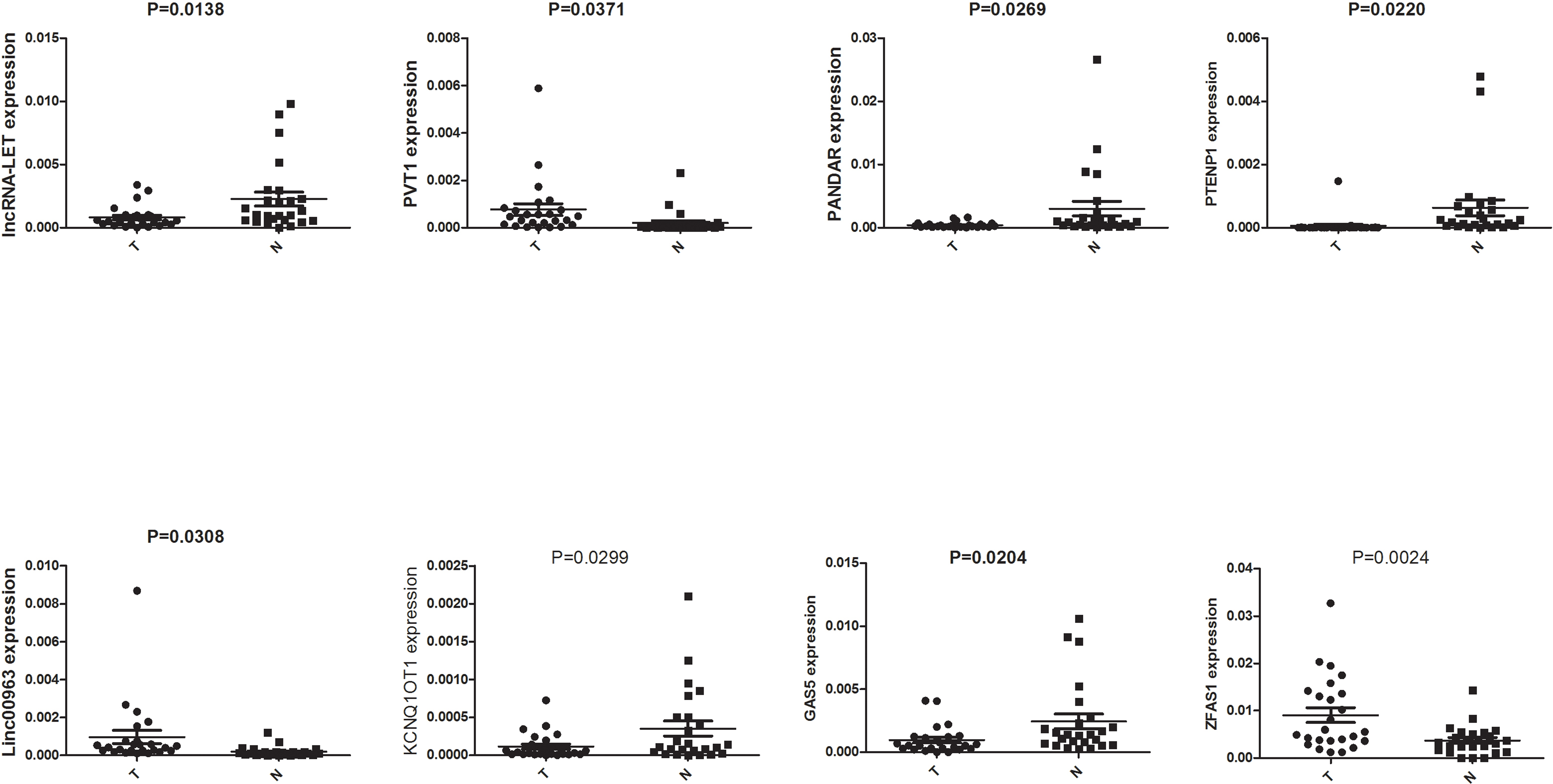

Supplement: Supplementary Figure S1 [file oncsis201548x1.tif]

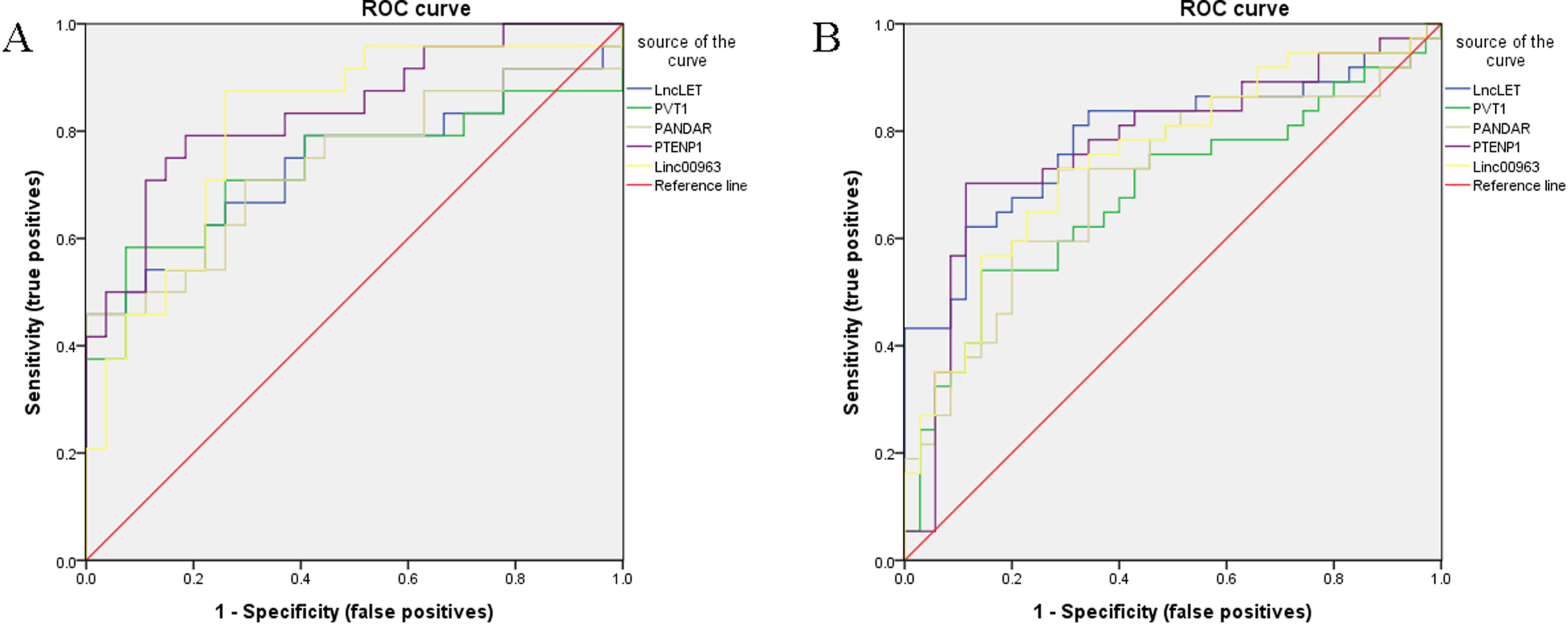

Supplement: Supplementary Figure S2 [file oncsis201548x2.tif]
