# Supplementary material for: A serum-circulating long noncoding RNA signature can discriminate between patients with clear cell renal cell carcinoma and healthy controls
Source: Oncogenesis. 2016 Feb 15;5(2):e192–. doi: 10.1038/oncsis.2015.48 (PMC5154346; doi:10.1038/oncsis.2015.48)
Supplement: Supplementary Figure Legends [file oncsis201548x6.doc]

Additional file 1 Fig. S1. Eight examples of the different expression of 82 lncRNAs in 25 cancerous tissues and paired adjacent non-tumorous specimens of patients.

Additional file 1 Fig. S2. The ROC curves of lncRNA-LET, PVT1, PANDAR, PTENP1 and linc00963 in the training group (A) and testing group (B).
